# Crystal structure of chlorido­(2-{1-[2-(4-chloro­phen­yl)hydrazin-1-yl­idene-κ*N*]eth­yl}pyridine-κ*N*)(η^5^-penta­methyl­cyclo­penta­dien­yl)rhodium(III) chloride

**DOI:** 10.1107/S205698901500184X

**Published:** 2015-02-07

**Authors:** Neelakandan Devika, Nandhagopal Raja, Subbiah Ananthalakshmi, Bruno Therrien

**Affiliations:** a Department of Chemistry, BIT Campus, Anna University, Tiruchirappalli 620 024, Tamil Nadu State, India; b Institut de Chimie, Université de Neuchâtel, Avenue de Bellevaux 51, CH-2000 Neuchâtel, Switzerland; c Department of Chemistry, Urumu Dhanalakshmi College, Tiruchirappalli 620 019, Tamil Nadu State, India

**Keywords:** crystal structure, rhodium(III) complex, penta­methyl­cyclo­penta­dien­yl, piano-stool geometry, N—H⋯Cl hydrogen bond

## Abstract

The cation of the title compound, [Rh(η^5^-C_5_Me_5_)Cl(C_13_H_12_ClN_3_)]Cl, adopts a typical piano-stool geometry. The complex is chiral at the metal and crystallizes as a racemate. Upon coordination, the hydrazinyl­idene­pyridine ligand is non-planar, an angle of 54.42 (7)° being observed between the pyridine ring and the aromatic ring of the [2-(4-chloro­phen­yl)hydrazin-1-yl­idene]ethyl group. In the crystal, a weak inter­ionic N—H⋯Cl hydrogen bond is observed.

## Abstract 2

The title compound, [Rh(η^5^-C_5_Me_5_)Cl(C_13_H_12_ClN_3_)]Cl, is chiral at the metal and crystallizes as a racemate. Upon coordination, the hydrazinyl­idene­pyridine ligand is non-planar, an angle of 54.42 (7)° being observed between the pyridine ring and the aromatic ring of the [2-(4-chloro­phen­yl)hydrazin-1-yl­idene]ethyl group.

## Chemical context   

Chiral-at-metal penta­methyl­cyclo­penta­dienyl rhodium complexes are popular catalysts in enanti­oselective reactions (Carmona *et al.*, 1999 ▸; Davies *et al.*, 2004 ▸). To obtain such chiral-at-metal complexes, a non-symmetrical bidentate ligand can be used. Among bidentate ligands, hydrazinyl­idene­pyridine derivatives are easy to synthesise (Liu *et al.*, 2002 ▸; Ghedini *et al.*, 2004 ▸; Marandi *et al.*, 2015 ▸), and when coupled to metal centers not only can they introduce chirality, but also they can generate biologically relevant complexes (Ghosh *et al.*, 2011 ▸, 2012 ▸). Herein, we present the synthesis and characterization of a chiral-at-metal penta­methyl­cyclo­pentadienyl rhodium(III) hydrazinyl­idene­pyridine complex, [Rh(η^5^-C_5_Me_5_)Cl(C_13_H_12_ClN_3_)]Cl.
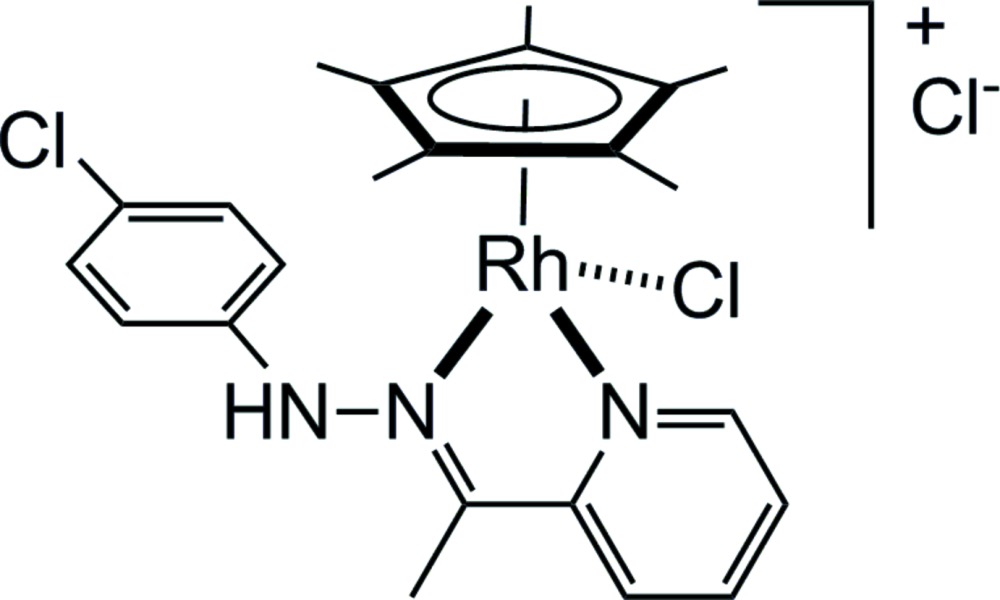


## Structural commentary   

**Figure 1:**
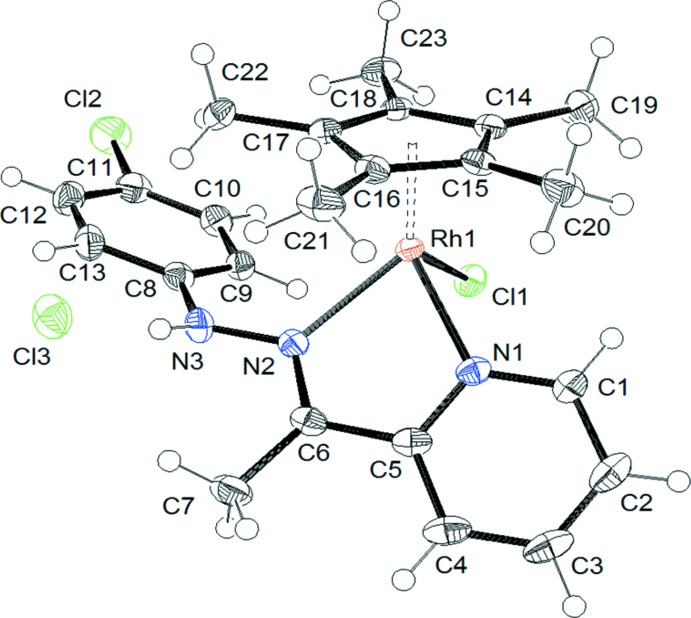
The mol­ecular structure of the title compound, with displacement ellipsoids drawn at the 50% probability level.

The mol­ecular structure of the title compound is presented in Fig. 1 ▸. The cationic complex adopts a typical piano-stool geometry and it is chiral at the metal centre. The salt crystallizes as a racemate in the ortho­rhom­bic space group *Pbca*. In the complex, the hydrazinyl­idene­pyridine ligand is *N,N-*coordinating, the *N*-hydrazono and the *N*-pyridine groups forming with the rhodium(III) atom a five-membered metalla­cycle. Upon coordination, the hydrazinyl­idene­pyridine ligand is non-planar, an angle of 54.42 (7)° being observed between the planes of pyridine and the benzene ring of the [(4-chloro­phen­yl)hydrazono]ethyl group. Otherwise, all geometrical data around the rhodium(III) atom are similar to those found in analogous *N*,*N*-chelated penta­methyl­cyclo­penta­dienyl rhodium complexes (Gupta *et al.*, 2011 ▸; Payne *et al.*, 2013 ▸).

## Supra­molecular features   

The N—H group of the hydrazinyl­idene­pyridine ligand inter­acts weakly with the counter-anion giving rise to a nearly linear hydrogen bond (Table 1 ▸). No significant C—H⋯π or π–π stacking inter­actions are observed.

## Synthesis and crystallization   

The title compound was synthesized by reacting one equivalent of [(η^5^-C_5_Me_5_)_2_Rh_2_(μ-Cl)_2_Cl_2_] (100 mg, 0.16 mmol) with two equivalents of 2-{1-[2-(4-chloro­phen­yl)hydrazono]eth­yl}pyridine (Liu *et al.*, 2002 ▸; 79 mg, 0.32 mmol) in methanol (25 ml), and the mixture was refluxed for 6 h. The solution turned from yellow to dark red. Then, the volume was reduced to 2 ml and diethyl ether was added to induce precipitation of a red–brown solid. After filtration, the solid was purified by column chromatography (silica gel, chloro­form/methanol 9.8:0.2 *v*/*v*). Crystals suitable for X-ray structure analysis were obtained by slow evaporation of a di­chloro­methane/*n*-pentane solution (1:1 *v*/*v*) containing the title compound. Yield: 80%. IR (KBr, ν, cm^−1^): 1592 (*s*, C=N). ^1^H NMR (400 MHz, CD_3_CN, 298 K): δ (p.p.m.) = 9.21 (*br s*, 1H, NH), 8.76 (*d*, ^3^ *J*_H-H_ = 5.6 Hz, 1H, H_ar_), 8.16 (*dd*, ^3^ *J*_H-H_ = 8.0 Hz, 1H, H_ar_), 8.01 (*d*, ^3^ *J*_H-H_ = 8.0 Hz, 1H, H_ar_), 7.77 (*dd*, ^3^ *J*_H-H_ = 6.8 Hz, 1H, H_ar_), 7.45 (*d*, ^3^ *J*_H-H_ = 8.8 Hz, 2H, H_ar_), 7.36 (*d*, ^3^ *J*_H-H_ = 8.8 Hz, 2H, H_ar_), 2.58 (*s*, 3H, CH_3_), 1.43 (*s*, 15H, C_5_Me_5_). MS (ESI positive mode): *m*/*z* 518.0 [*M* − Cl]^+^.

## Refinement   

Crystal data, data collection and structure refinement details are summarized in Table 2 ▸. Except for the N-bound H atom, which was refined freely, all hydrogen atoms were included in calculated positions and treated as riding atoms using *SHELXL97* default parameters, with C—H = 0.93 Å for C_arom_ and 0.96 Å for CH_3_, and with *U*_iso_(H) = 1.2 *U*_eq_(C) or 1.5 *U*_eq_(C) for methyl H atoms.

## Supplementary Material

Crystal structure: contains datablock(s) I, global. DOI: 10.1107/S205698901500184X/rz5146sup1.cif

Structure factors: contains datablock(s) I. DOI: 10.1107/S205698901500184X/rz5146Isup2.hkl

CCDC reference: 1045840

Additional supporting information:  crystallographic information; 3D view; checkCIF report

## Figures and Tables

**Table 1:** Hydrogen-bond geometry (, )

*D*H*A*	*D*H	H*A*	*D* *A*	*D*H*A*
N3H3*N*Cl3	0.83(3)	2.27(3)	3.087(2)	171(3)

**Table 2:** Experimental details

Crystal data	
Chemical formula	[Rh(C_10_H_15_)Cl(C_13_H_12_ClN_3_)]Cl
*M*_r_	554.74
Crystal system, space group	Orthorhombic, *P* *b* *c* *a*
Temperature (K)	173
*a*, *b*, *c* ()	13.0774(5), 13.4537(5), 26.5153(9)
*V* (^3^)	4665.1(3)
*Z*	8
Radiation type	Mo *K*
(mm^1^)	1.09
Crystal size (mm)	0.21 0.20 0.13

Data collection	
Diffractometer	STOE *IPDS* diffractometer
Absorption correction	Empirical (using intensity measurements) (*DIFABS*; Walker Stuart, 1983 ▸)
*T*_min_, *T*_max_	0.629, 0.890
No. of measured, independent and observed [*I* > 2(*I*)] reflections	82717, 6320, 4619
*R*_int_	0.074
(sin /)_max_ (^1^)	0.687

Refinement	
*R*[*F* ^2^ > 2(*F* ^2^)], *wR*(*F* ^2^), *S*	0.032, 0.054, 0.96
No. of reflections	6320
No. of parameters	281
H-atom treatment	H atoms treated by a mixture of independent and constrained refinement
_max_, _min_ (e ^3^)	0.48, 0.62

Computer programs: *IPDS* *EXPOSE* (Stoe Cie, 2000 ▸), *IPDS* *CELL* (Stoe Cie, 2000 ▸), *IPDS* *INTEGRATE* (Stoe Cie, 2000 ▸), *SHELXS97* (Sheldrick, 2008 ▸), *SHELXL97* (Sheldrick, 2008 ▸), *ORTEP-32* (Farrugia, 2012 ▸).

## supplementary crystallographic information

### Crystal data

**Table:**

[Rh(C_10_H_15_)Cl(C_13_H_12_ClN_3_)]Cl	*F*(000) = 2256
*M**_r_* = 554.74	*D*_x_ = 1.580 Mg m^−^^3^
Orthorhombic, *P**b**c**a*	Mo *K*α radiation, λ = 0.71073 Å
Hall symbol: -P 2ac 2ab	Cell parameters from 8000 reflections
*a* = 13.0774 (5) Å	θ = 2.4–28.9°
*b* = 13.4537 (5) Å	µ = 1.09 mm^−^^1^
*c* = 26.5153 (9) Å	*T* = 173 K
*V* = 4665.1 (3) Å^3^	Rod, yellow
*Z* = 8	0.21 × 0.20 × 0.13 mm

### Data collection

**Table:**

STOE IPDS diffractometer	6320 independent reflections
Radiation source: fine-focus sealed tube	4619 reflections with *I* > 2σ(*I*)
Graphite monochromator	*R*_int_ = 0.074
Detector resolution: 0.81 pixels mm^-1^	θ_max_ = 29.3°, θ_min_ = 2.2°
phi oscillation scans	*h* = −17→17
Absorption correction: empirical (using intensity measurements) (*DIFABS*; Walker & Stuart, 1983)	*k* = −18→18
*T*_min_ = 0.629, *T*_max_ = 0.890	*l* = −36→36
82717 measured reflections	

### Refinement

**Table:**

Refinement on *F*^2^	Primary atom site location: structure-invariant direct methods
Least-squares matrix: full	Secondary atom site location: difference Fourier map
*R*[*F*^2^ > 2σ(*F*^2^)] = 0.032	Hydrogen site location: inferred from neighbouring sites
*wR*(*F*^2^) = 0.054	H atoms treated by a mixture of independent and constrained refinement
*S* = 0.96	*w* = 1/[σ^2^(*F*_o_^2^) + (0.0236*P*)^2^] where *P* = (*F*_o_^2^ + 2*F*_c_^2^)/3
6320 reflections	(Δ/σ)_max_ = 0.005
281 parameters	Δρ_max_ = 0.48 e Å^−^^3^
0 restraints	Δρ_min_ = −0.62 e Å^−^^3^

### Special details

**Table:**

Experimental. A crystal was mounted at 173 K on a Stoe Image Plate Diffraction System (Stoe & Cie, 2000) using Mo *K*α graphite monochromated radiation. Image plate distance 100 mm, φ oscillation scans 0 - 180°, step Δφ = 0.8°, 5 minutes per frame.
Geometry. All e.s.d.'s (except the e.s.d. in the dihedral angle between two l.s. planes) are estimated using the full covariance matrix. The cell e.s.d.'s are taken into account individually in the estimation of e.s.d.'s in distances, angles and torsion angles; correlations between e.s.d.'s in cell parameters are only used when they are defined by crystal symmetry. An approximate (isotropic) treatment of cell e.s.d.'s is used for estimating e.s.d.'s involving l.s. planes.
Refinement. Refinement of *F*^2^ against ALL reflections. The weighted *R*-factor *wR* and goodness of fit *S* are based on *F*^2^, conventional *R*-factors *R* are based on *F*, with *F* set to zero for negative *F*^2^. The threshold expression of *F*^2^ > σ(*F*^2^) is used only for calculating *R*-factors(gt) *etc*. and is not relevant to the choice of reflections for refinement. *R*-factors based on *F*^2^ are statistically about twice as large as those based on *F*, and *R*- factors based on ALL data will be even larger.

### Fractional atomic coordinates and isotropic or equivalent isotropic displacement parameters (Å^2^)

**Table:**

	*x*	*y*	*z*	*U*_iso_*/*U*_eq_	
C1	0.57249 (18)	−0.00756 (18)	0.60832 (10)	0.0279 (5)	
H1	0.5871	0.0234	0.5778	0.034*	
C2	0.6422 (2)	−0.07526 (19)	0.62774 (11)	0.0355 (6)	
H2	0.7029	−0.0886	0.6107	0.043*	
C3	0.6205 (2)	−0.12238 (18)	0.67247 (10)	0.0350 (6)	
H3	0.6658	−0.1687	0.6859	0.042*	
C4	0.5302 (2)	−0.09975 (19)	0.69720 (9)	0.0312 (5)	
H4	0.5145	−0.1304	0.7277	0.037*	
C5	0.46296 (18)	−0.03094 (16)	0.67623 (8)	0.0218 (5)	
C6	0.36119 (19)	−0.01036 (16)	0.69694 (8)	0.0217 (5)	
C7	0.3320 (2)	−0.04681 (19)	0.74812 (9)	0.0315 (6)	
H7A	0.2787	−0.0055	0.7616	0.047*	
H7B	0.3905	−0.0443	0.7700	0.047*	
H7C	0.3080	−0.1141	0.7457	0.047*	
C8	0.11641 (18)	0.05175 (16)	0.65302 (8)	0.0214 (5)	
C9	0.11770 (18)	0.00803 (16)	0.60562 (9)	0.0228 (4)	
H9	0.1771	−0.0224	0.5940	0.027*	
C10	0.03097 (19)	0.00944 (18)	0.57538 (9)	0.0263 (5)	
H10	0.0324	−0.0192	0.5435	0.032*	
C11	−0.05724 (18)	0.05360 (19)	0.59299 (9)	0.0282 (6)	
C12	−0.06171 (19)	0.09489 (19)	0.64099 (10)	0.0310 (6)	
H12	−0.1222	0.1227	0.6529	0.037*	
C13	0.02538 (18)	0.09408 (18)	0.67088 (9)	0.0277 (5)	
H13	0.0234	0.1218	0.7030	0.033*	
C14	0.41151 (18)	0.21684 (16)	0.54714 (8)	0.0185 (4)	
C15	0.46438 (17)	0.23872 (16)	0.59274 (8)	0.0197 (5)	
C16	0.39032 (18)	0.25436 (15)	0.63199 (8)	0.0203 (5)	
C17	0.29044 (17)	0.24955 (15)	0.60875 (9)	0.0206 (5)	
C18	0.30297 (17)	0.22316 (15)	0.55724 (8)	0.0186 (4)	
C19	0.4574 (2)	0.19802 (19)	0.49645 (8)	0.0277 (5)	
H19A	0.4671	0.2601	0.4792	0.042*	
H19B	0.4124	0.1565	0.4771	0.042*	
H19C	0.5221	0.1654	0.5004	0.042*	
C20	0.57832 (18)	0.2459 (2)	0.59914 (11)	0.0314 (6)	
H20A	0.6115	0.2052	0.5743	0.047*	
H20B	0.5969	0.2235	0.6323	0.047*	
H20C	0.5994	0.3138	0.5949	0.047*	
C21	0.4125 (2)	0.28192 (18)	0.68547 (9)	0.0310 (6)	
H21A	0.4770	0.2540	0.6954	0.046*	
H21B	0.3594	0.2565	0.7069	0.046*	
H21C	0.4153	0.3530	0.6885	0.046*	
C22	0.1914 (2)	0.27337 (17)	0.63382 (10)	0.0288 (5)	
H22A	0.1771	0.3430	0.6302	0.043*	
H22B	0.1957	0.2570	0.6690	0.043*	
H22C	0.1376	0.2354	0.6185	0.043*	
C23	0.22078 (19)	0.20957 (18)	0.51908 (9)	0.0272 (5)	
H23A	0.1577	0.1938	0.5358	0.041*	
H23B	0.2389	0.1563	0.4967	0.041*	
H23C	0.2126	0.2698	0.5001	0.041*	
Cl1	0.34471 (4)	−0.02319 (4)	0.54150 (2)	0.02355 (12)	
Cl2	−0.16517 (5)	0.05548 (6)	0.55360 (3)	0.04123 (17)	
Cl3	0.19592 (5)	0.18619 (5)	0.77896 (2)	0.03405 (14)	
N1	0.48505 (14)	0.01482 (14)	0.63192 (7)	0.0207 (4)	
N2	0.29842 (14)	0.03410 (13)	0.66601 (7)	0.0184 (4)	
N3	0.20095 (15)	0.05148 (15)	0.68473 (7)	0.0229 (4)	
H3N	0.199 (2)	0.082 (2)	0.7119 (11)	0.036 (8)*	
Rh1	0.371080 (13)	0.107403 (11)	0.602805 (6)	0.01552 (4)	

### Atomic displacement parameters (Å^2^)

**Table:**

	*U*^11^	*U*^22^	*U*^33^	*U*^12^	*U*^13^	*U*^23^
C1	0.0221 (12)	0.0290 (12)	0.0328 (13)	0.0055 (10)	0.0013 (11)	0.0033 (11)
C2	0.0236 (14)	0.0349 (13)	0.0481 (15)	0.0099 (11)	−0.0049 (12)	−0.0034 (12)
C3	0.0299 (14)	0.0300 (13)	0.0453 (14)	0.0086 (12)	−0.0162 (13)	0.0007 (11)
C4	0.0368 (14)	0.0278 (12)	0.0289 (12)	0.0045 (12)	−0.0134 (11)	0.0037 (11)
C5	0.0260 (13)	0.0187 (11)	0.0207 (11)	−0.0015 (9)	−0.0078 (9)	−0.0006 (9)
C6	0.0296 (13)	0.0185 (10)	0.0169 (10)	−0.0028 (10)	−0.0025 (10)	−0.0004 (8)
C7	0.0438 (16)	0.0298 (13)	0.0207 (12)	0.0022 (12)	−0.0004 (11)	0.0060 (10)
C8	0.0220 (12)	0.0184 (10)	0.0239 (10)	−0.0042 (9)	0.0025 (10)	0.0013 (8)
C9	0.0213 (12)	0.0232 (10)	0.0240 (10)	−0.0001 (9)	0.0025 (10)	0.0005 (10)
C10	0.0263 (13)	0.0288 (12)	0.0238 (12)	−0.0033 (10)	0.0001 (10)	−0.0005 (10)
C11	0.0194 (12)	0.0308 (13)	0.0345 (15)	−0.0045 (10)	−0.0032 (10)	0.0075 (10)
C12	0.0220 (12)	0.0282 (13)	0.0427 (14)	0.0001 (11)	0.0081 (11)	0.0021 (11)
C13	0.0257 (12)	0.0289 (13)	0.0286 (12)	−0.0009 (10)	0.0075 (10)	−0.0023 (10)
C14	0.0233 (11)	0.0158 (10)	0.0163 (10)	0.0010 (9)	−0.0006 (9)	0.0015 (8)
C15	0.0224 (11)	0.0159 (10)	0.0209 (12)	−0.0034 (9)	−0.0014 (9)	0.0041 (8)
C16	0.0271 (14)	0.0159 (9)	0.0178 (10)	−0.0011 (9)	−0.0007 (9)	−0.0006 (8)
C17	0.0239 (11)	0.0134 (9)	0.0245 (12)	0.0018 (8)	0.0017 (10)	0.0020 (9)
C18	0.0200 (11)	0.0146 (10)	0.0212 (11)	0.0015 (9)	−0.0015 (9)	0.0030 (8)
C19	0.0317 (14)	0.0321 (13)	0.0192 (11)	0.0044 (11)	0.0045 (10)	0.0011 (10)
C20	0.0235 (12)	0.0361 (13)	0.0345 (13)	−0.0079 (11)	−0.0043 (12)	0.0089 (12)
C21	0.0482 (16)	0.0258 (12)	0.0190 (11)	0.0002 (11)	−0.0051 (11)	−0.0021 (10)
C22	0.0316 (14)	0.0191 (11)	0.0358 (14)	0.0067 (10)	0.0113 (11)	0.0010 (10)
C23	0.0270 (14)	0.0261 (12)	0.0285 (12)	0.0010 (10)	−0.0083 (10)	0.0028 (10)
Cl1	0.0249 (3)	0.0202 (2)	0.0255 (3)	0.0013 (2)	0.0007 (2)	−0.0065 (2)
Cl2	0.0240 (3)	0.0549 (4)	0.0448 (4)	−0.0029 (3)	−0.0080 (3)	0.0091 (3)
Cl3	0.0422 (4)	0.0344 (3)	0.0256 (3)	−0.0010 (3)	0.0048 (3)	−0.0084 (2)
N1	0.0191 (10)	0.0193 (9)	0.0236 (10)	0.0007 (8)	−0.0038 (8)	0.0007 (8)
N2	0.0208 (10)	0.0158 (9)	0.0186 (9)	−0.0012 (7)	0.0004 (8)	−0.0013 (7)
N3	0.0221 (10)	0.0279 (11)	0.0188 (9)	−0.0007 (9)	0.0027 (8)	−0.0045 (8)
Rh1	0.01625 (7)	0.01479 (6)	0.01552 (6)	0.00163 (7)	−0.00072 (8)	0.00038 (7)

### Geometric parameters (Å, º)

**Table:**

C1—N1	1.338 (3)	C15—C16	1.437 (3)
C1—C2	1.388 (3)	C15—C20	1.503 (3)
C1—H1	0.9300	C15—Rh1	2.164 (2)
C2—C3	1.375 (4)	C16—C17	1.446 (3)
C2—H2	0.9300	C16—C21	1.494 (3)
C3—C4	1.385 (4)	C16—Rh1	2.138 (2)
C3—H3	0.9300	C17—C18	1.421 (3)
C4—C5	1.392 (3)	C17—C22	1.491 (3)
C4—H4	0.9300	C17—Rh1	2.190 (2)
C5—N1	1.357 (3)	C18—C23	1.487 (3)
C5—C6	1.466 (3)	C18—Rh1	2.163 (2)
C6—N2	1.306 (3)	C19—H19A	0.9600
C6—C7	1.493 (3)	C19—H19B	0.9600
C7—H7A	0.9600	C19—H19C	0.9600
C7—H7B	0.9600	C20—H20A	0.9600
C7—H7C	0.9600	C20—H20B	0.9600
C8—C9	1.388 (3)	C20—H20C	0.9600
C8—N3	1.389 (3)	C21—H21A	0.9600
C8—C13	1.402 (3)	C21—H21B	0.9600
C9—C10	1.389 (3)	C21—H21C	0.9600
C9—H9	0.9300	C22—H22A	0.9600
C10—C11	1.379 (3)	C22—H22B	0.9600
C10—H10	0.9300	C22—H22C	0.9600
C11—C12	1.390 (4)	C23—H23A	0.9600
C11—Cl2	1.756 (2)	C23—H23B	0.9600
C12—C13	1.388 (4)	C23—H23C	0.9600
C12—H12	0.9300	Cl1—Rh1	2.4183 (6)
C13—H13	0.9300	N1—Rh1	2.0902 (18)
C14—C15	1.424 (3)	N2—N3	1.388 (3)
C14—C18	1.447 (3)	N2—Rh1	2.1643 (18)
C14—C19	1.493 (3)	N3—H3N	0.83 (3)
C14—Rh1	2.151 (2)		
			
N1—C1—C2	122.4 (2)	C14—C18—Rh1	69.95 (12)
N1—C1—H1	118.8	C23—C18—Rh1	126.08 (15)
C2—C1—H1	118.8	C14—C19—H19A	109.5
C3—C2—C1	119.1 (3)	C14—C19—H19B	109.5
C3—C2—H2	120.4	H19A—C19—H19B	109.5
C1—C2—H2	120.4	C14—C19—H19C	109.5
C2—C3—C4	118.9 (2)	H19A—C19—H19C	109.5
C2—C3—H3	120.6	H19B—C19—H19C	109.5
C4—C3—H3	120.6	C15—C20—H20A	109.5
C3—C4—C5	119.7 (2)	C15—C20—H20B	109.5
C3—C4—H4	120.2	H20A—C20—H20B	109.5
C5—C4—H4	120.2	C15—C20—H20C	109.5
N1—C5—C4	120.9 (2)	H20A—C20—H20C	109.5
N1—C5—C6	115.58 (19)	H20B—C20—H20C	109.5
C4—C5—C6	123.3 (2)	C16—C21—H21A	109.5
N2—C6—C5	114.96 (19)	C16—C21—H21B	109.5
N2—C6—C7	124.1 (2)	H21A—C21—H21B	109.5
C5—C6—C7	120.7 (2)	C16—C21—H21C	109.5
C6—C7—H7A	109.5	H21A—C21—H21C	109.5
C6—C7—H7B	109.5	H21B—C21—H21C	109.5
H7A—C7—H7B	109.5	C17—C22—H22A	109.5
C6—C7—H7C	109.5	C17—C22—H22B	109.5
H7A—C7—H7C	109.5	H22A—C22—H22B	109.5
H7B—C7—H7C	109.5	C17—C22—H22C	109.5
C9—C8—N3	122.5 (2)	H22A—C22—H22C	109.5
C9—C8—C13	119.2 (2)	H22B—C22—H22C	109.5
N3—C8—C13	118.2 (2)	C18—C23—H23A	109.5
C8—C9—C10	120.5 (2)	C18—C23—H23B	109.5
C8—C9—H9	119.8	H23A—C23—H23B	109.5
C10—C9—H9	119.8	C18—C23—H23C	109.5
C11—C10—C9	119.6 (2)	H23A—C23—H23C	109.5
C11—C10—H10	120.2	H23B—C23—H23C	109.5
C9—C10—H10	120.2	C1—N1—C5	119.0 (2)
C10—C11—C12	121.2 (2)	C1—N1—Rh1	124.81 (16)
C10—C11—Cl2	118.5 (2)	C5—N1—Rh1	115.99 (15)
C12—C11—Cl2	120.3 (2)	C6—N2—N3	115.47 (18)
C13—C12—C11	119.0 (2)	C6—N2—Rh1	114.78 (15)
C13—C12—H12	120.5	N3—N2—Rh1	127.12 (14)
C11—C12—H12	120.5	N2—N3—C8	121.01 (19)
C12—C13—C8	120.5 (2)	N2—N3—H3N	115 (2)
C12—C13—H13	119.8	C8—N3—H3N	120 (2)
C8—C13—H13	119.8	N1—Rh1—C16	109.48 (8)
C15—C14—C18	107.88 (19)	N1—Rh1—C14	119.08 (8)
C15—C14—C19	127.2 (2)	C16—Rh1—C14	65.57 (8)
C18—C14—C19	124.8 (2)	N1—Rh1—C18	158.28 (8)
C15—C14—Rh1	71.22 (12)	C16—Rh1—C18	65.47 (8)
C18—C14—Rh1	70.85 (12)	C14—Rh1—C18	39.20 (9)
C19—C14—Rh1	126.89 (16)	N1—Rh1—C15	97.47 (8)
C14—C15—C16	108.55 (19)	C16—Rh1—C15	39.03 (8)
C14—C15—C20	126.2 (2)	C14—Rh1—C15	38.53 (8)
C16—C15—C20	125.2 (2)	C18—Rh1—C15	64.87 (8)
C14—C15—Rh1	70.25 (12)	N1—Rh1—N2	75.85 (7)
C16—C15—Rh1	69.52 (12)	C16—Rh1—N2	101.11 (7)
C20—C15—Rh1	126.73 (16)	C14—Rh1—N2	162.04 (8)
C15—C16—C17	107.07 (18)	C18—Rh1—N2	125.44 (8)
C15—C16—C21	126.4 (2)	C15—Rh1—N2	135.66 (7)
C17—C16—C21	126.2 (2)	N1—Rh1—C17	146.86 (8)
C15—C16—Rh1	71.45 (12)	C16—Rh1—C17	39.01 (8)
C17—C16—Rh1	72.42 (12)	C14—Rh1—C17	64.52 (8)
C21—C16—Rh1	126.57 (16)	C18—Rh1—C17	38.09 (8)
C18—C17—C16	108.47 (19)	C15—Rh1—C17	64.36 (8)
C18—C17—C22	125.6 (2)	N2—Rh1—C17	97.52 (8)
C16—C17—C22	125.8 (2)	N1—Rh1—Cl1	85.24 (5)
C18—C17—Rh1	69.94 (12)	C16—Rh1—Cl1	158.74 (6)
C16—C17—Rh1	68.57 (12)	C14—Rh1—Cl1	94.08 (6)
C22—C17—Rh1	129.66 (16)	C18—Rh1—Cl1	95.10 (6)
C17—C18—C14	107.81 (19)	C15—Rh1—Cl1	126.21 (6)
C17—C18—C23	127.0 (2)	N2—Rh1—Cl1	97.29 (5)
C14—C18—C23	125.2 (2)	C17—Rh1—Cl1	127.90 (6)
C17—C18—Rh1	71.97 (12)		
			
N1—C1—C2—C3	1.0 (4)	C15—C16—Rh1—N2	156.22 (12)
C1—C2—C3—C4	−0.9 (4)	C17—C16—Rh1—N2	−88.24 (13)
C2—C3—C4—C5	0.6 (4)	C21—C16—Rh1—N2	34.2 (2)
C3—C4—C5—N1	−0.4 (4)	C15—C16—Rh1—C17	−115.54 (17)
C3—C4—C5—C6	173.4 (2)	C21—C16—Rh1—C17	122.5 (3)
N1—C5—C6—N2	12.0 (3)	C15—C16—Rh1—Cl1	−54.3 (2)
C4—C5—C6—N2	−162.0 (2)	C17—C16—Rh1—Cl1	61.2 (2)
N1—C5—C6—C7	−173.1 (2)	C21—C16—Rh1—Cl1	−176.30 (14)
C4—C5—C6—C7	12.9 (3)	C15—C14—Rh1—N1	−62.63 (15)
N3—C8—C9—C10	179.5 (2)	C18—C14—Rh1—N1	179.89 (11)
C13—C8—C9—C10	2.4 (3)	C19—C14—Rh1—N1	60.3 (2)
C8—C9—C10—C11	−0.8 (3)	C15—C14—Rh1—C16	36.94 (13)
C9—C10—C11—C12	−1.5 (4)	C18—C14—Rh1—C16	−80.53 (14)
C9—C10—C11—Cl2	179.27 (18)	C19—C14—Rh1—C16	159.8 (2)
C10—C11—C12—C13	2.0 (4)	C15—C14—Rh1—C18	117.47 (18)
Cl2—C11—C12—C13	−178.73 (19)	C19—C14—Rh1—C18	−119.6 (3)
C11—C12—C13—C8	−0.3 (4)	C18—C14—Rh1—C15	−117.47 (18)
C9—C8—C13—C12	−1.9 (3)	C19—C14—Rh1—C15	122.9 (3)
N3—C8—C13—C12	−179.0 (2)	C15—C14—Rh1—N2	81.3 (3)
C18—C14—C15—C16	2.6 (2)	C18—C14—Rh1—N2	−36.1 (3)
C19—C14—C15—C16	178.3 (2)	C19—C14—Rh1—N2	−155.8 (2)
Rh1—C14—C15—C16	−59.16 (15)	C15—C14—Rh1—C17	80.15 (14)
C18—C14—C15—C20	−176.6 (2)	C18—C14—Rh1—C17	−37.33 (12)
C19—C14—C15—C20	−0.9 (4)	C19—C14—Rh1—C17	−157.0 (2)
Rh1—C14—C15—C20	121.6 (2)	C15—C14—Rh1—Cl1	−149.45 (12)
C18—C14—C15—Rh1	61.73 (15)	C18—C14—Rh1—Cl1	93.08 (12)
C19—C14—C15—Rh1	−122.5 (2)	C19—C14—Rh1—Cl1	−26.6 (2)
C14—C15—C16—C17	−4.5 (2)	C17—C18—Rh1—N1	−117.7 (2)
C20—C15—C16—C17	174.7 (2)	C14—C18—Rh1—N1	−0.3 (3)
Rh1—C15—C16—C17	−64.13 (14)	C23—C18—Rh1—N1	119.2 (2)
C14—C15—C16—C21	−178.1 (2)	C17—C18—Rh1—C16	−36.65 (13)
C20—C15—C16—C21	1.1 (4)	C14—C18—Rh1—C16	80.81 (14)
Rh1—C15—C16—C21	122.2 (2)	C23—C18—Rh1—C16	−159.7 (2)
C14—C15—C16—Rh1	59.61 (15)	C17—C18—Rh1—C14	−117.46 (18)
C20—C15—C16—Rh1	−121.2 (2)	C23—C18—Rh1—C14	119.5 (2)
C15—C16—C17—C18	4.8 (2)	C17—C18—Rh1—C15	−79.84 (14)
C21—C16—C17—C18	178.4 (2)	C14—C18—Rh1—C15	37.62 (12)
Rh1—C16—C17—C18	−58.69 (14)	C23—C18—Rh1—C15	157.1 (2)
C15—C16—C17—C22	−172.3 (2)	C17—C18—Rh1—N2	49.64 (15)
C21—C16—C17—C22	1.3 (4)	C14—C18—Rh1—N2	167.10 (11)
Rh1—C16—C17—C22	124.2 (2)	C23—C18—Rh1—N2	−73.4 (2)
C15—C16—C17—Rh1	63.49 (14)	C14—C18—Rh1—C17	117.46 (18)
C21—C16—C17—Rh1	−122.9 (2)	C23—C18—Rh1—C17	−123.0 (3)
C16—C17—C18—C14	−3.3 (2)	C17—C18—Rh1—Cl1	152.34 (12)
C22—C17—C18—C14	173.9 (2)	C14—C18—Rh1—Cl1	−90.20 (12)
Rh1—C17—C18—C14	−61.10 (15)	C23—C18—Rh1—Cl1	29.3 (2)
C16—C17—C18—C23	179.9 (2)	C14—C15—Rh1—N1	128.49 (13)
C22—C17—C18—C23	−3.0 (4)	C16—C15—Rh1—N1	−111.85 (13)
Rh1—C17—C18—C23	122.0 (2)	C20—C15—Rh1—N1	7.5 (2)
C16—C17—C18—Rh1	57.85 (14)	C14—C15—Rh1—C16	−119.66 (18)
C22—C17—C18—Rh1	−125.0 (2)	C20—C15—Rh1—C16	119.3 (3)
C15—C14—C18—C17	0.4 (2)	C16—C15—Rh1—C14	119.66 (18)
C19—C14—C18—C17	−175.4 (2)	C20—C15—Rh1—C14	−121.0 (3)
Rh1—C14—C18—C17	62.40 (15)	C14—C15—Rh1—C18	−38.27 (13)
C15—C14—C18—C23	177.4 (2)	C16—C15—Rh1—C18	81.39 (14)
C19—C14—C18—C23	1.5 (4)	C20—C15—Rh1—C18	−159.3 (2)
Rh1—C14—C18—C23	−120.7 (2)	C14—C15—Rh1—N2	−154.14 (12)
C15—C14—C18—Rh1	−61.96 (15)	C16—C15—Rh1—N2	−34.48 (17)
C19—C14—C18—Rh1	122.2 (2)	C20—C15—Rh1—N2	84.8 (2)
C2—C1—N1—C5	−0.7 (4)	C14—C15—Rh1—C17	−80.61 (14)
C2—C1—N1—Rh1	−175.13 (19)	C16—C15—Rh1—C17	39.05 (13)
C4—C5—N1—C1	0.4 (3)	C20—C15—Rh1—C17	158.4 (2)
C6—C5—N1—C1	−173.8 (2)	C14—C15—Rh1—Cl1	38.93 (15)
C4—C5—N1—Rh1	175.32 (17)	C16—C15—Rh1—Cl1	158.59 (10)
C6—C5—N1—Rh1	1.1 (2)	C20—C15—Rh1—Cl1	−82.1 (2)
C5—C6—N2—N3	178.27 (18)	C6—N2—Rh1—N1	14.84 (15)
C7—C6—N2—N3	3.5 (3)	N3—N2—Rh1—N1	175.49 (18)
C5—C6—N2—Rh1	−18.7 (2)	C6—N2—Rh1—C16	−92.67 (16)
C7—C6—N2—Rh1	166.52 (18)	N3—N2—Rh1—C16	67.98 (18)
C6—N2—N3—C8	−148.6 (2)	C6—N2—Rh1—C14	−133.1 (2)
Rh1—N2—N3—C8	50.9 (3)	N3—N2—Rh1—C14	27.5 (3)
C9—C8—N3—N2	19.0 (3)	C6—N2—Rh1—C18	−160.37 (15)
C13—C8—N3—N2	−163.9 (2)	N3—N2—Rh1—C18	0.3 (2)
C1—N1—Rh1—C16	−96.4 (2)	C6—N2—Rh1—C15	−71.37 (19)
C5—N1—Rh1—C16	89.00 (16)	N3—N2—Rh1—C15	89.28 (19)
C1—N1—Rh1—C14	−24.2 (2)	C6—N2—Rh1—C17	−132.06 (16)
C5—N1—Rh1—C14	161.22 (15)	N3—N2—Rh1—C17	28.58 (18)
C1—N1—Rh1—C18	−24.0 (3)	C6—N2—Rh1—Cl1	98.03 (15)
C5—N1—Rh1—C18	161.41 (19)	N3—N2—Rh1—Cl1	−101.32 (16)
C1—N1—Rh1—C15	−58.1 (2)	C18—C17—Rh1—N1	143.18 (15)
C5—N1—Rh1—C15	127.31 (16)	C16—C17—Rh1—N1	22.8 (2)
C1—N1—Rh1—N2	166.6 (2)	C22—C17—Rh1—N1	−96.6 (2)
C5—N1—Rh1—N2	−7.99 (15)	C18—C17—Rh1—C16	120.37 (18)
C1—N1—Rh1—C17	−111.4 (2)	C22—C17—Rh1—C16	−119.4 (3)
C5—N1—Rh1—C17	74.0 (2)	C18—C17—Rh1—C14	38.40 (13)
C1—N1—Rh1—Cl1	67.84 (19)	C16—C17—Rh1—C14	−81.97 (13)
C5—N1—Rh1—Cl1	−106.75 (15)	C22—C17—Rh1—C14	158.6 (2)
C15—C16—Rh1—N1	77.45 (13)	C16—C17—Rh1—C18	−120.37 (18)
C17—C16—Rh1—N1	−167.01 (12)	C22—C17—Rh1—C18	120.2 (3)
C21—C16—Rh1—N1	−44.5 (2)	C18—C17—Rh1—C15	81.31 (14)
C15—C16—Rh1—C14	−36.48 (12)	C16—C17—Rh1—C15	−39.07 (12)
C17—C16—Rh1—C14	79.06 (13)	C22—C17—Rh1—C15	−158.5 (2)
C21—C16—Rh1—C14	−158.5 (2)	C18—C17—Rh1—N2	−141.23 (13)
C15—C16—Rh1—C18	−79.73 (13)	C16—C17—Rh1—N2	98.39 (12)
C17—C16—Rh1—C18	35.81 (12)	C22—C17—Rh1—N2	−21.0 (2)
C21—C16—Rh1—C18	158.3 (2)	C18—C17—Rh1—Cl1	−35.87 (15)
C17—C16—Rh1—C15	115.54 (17)	C16—C17—Rh1—Cl1	−156.25 (10)
C21—C16—Rh1—C15	−122.0 (3)	C22—C17—Rh1—Cl1	84.3 (2)

### Hydrogen-bond geometry (Å, º)

**Table:**

*D*—H···*A*	*D*—H	H···*A*	*D*···*A*	*D*—H···*A*
N3—H3*N*···Cl3	0.83 (3)	2.27 (3)	3.087 (2)	171 (3)
